# Carbon Nanofibers Heavy Laden with Li_3_V_2_(PO_4_)_3_ Particles Featuring Superb Kinetics for High‐Power Lithium Ion Battery

**DOI:** 10.1002/advs.201700128

**Published:** 2017-05-12

**Authors:** Jeongyim Shin, Junghoon Yang, Chernov Sergey, Min‐Sang Song, Yong‐Mook Kang

**Affiliations:** ^1^ Department of Energy and Materials Engineering Dongguk University Seoul 100‐715 Republic of Korea; ^2^ Energy Material Lab Material Research Center Samsung Advanced Institute of Technology Samsung Electronics 130 Samsung‐ro Yeongtong‐gu, Suwon‐si Gyeonggi‐do 16678 Republic of Korea

**Keywords:** carbon nanofibers, Li_3_V_2_(PO_4_)_3_, lithium‐ion batteries, Ostwald ripening, unique structures

## Abstract

Fast lithium ion and electron transport inside electrode materials are essential to realize its superb electrochemical performances for lithium rechargeable batteries. Herein, a distinctive structure of cathode material is proposed, which can simultaneously satisfy these requirements. Nanosized Li_3_V_2_(PO_4_)_3_ (LVP) particles can be successfully grown up on the carbon nanofiber via electrospinning method followed by a controlled heat‐treatment. Herein, LVP particles are anchored onto the surface of carbon nanofiber, and with this growing process, the size of LVP particles as well as the thickness of carbon nanofiber can be regulated together. The morphological features of this composite structure enable not only direct contact between electrolytes and LVP particles that can enhance lithium ion diffusivity, but also fast electron transport through 1D carbon network along nanofibers simultaneously. Finally, it is demonstrated that this unique structure is an ideal one to realize high electron transport and ion diffusivity together, which are essential for enhancing the electrochemical performances of electrode materials.

## 1 Introduction

In the current society, rechargeable lithium‐ion batteries (LIBs) have been considered as one of the most important energy storage systems since they were invented.^1^, ^2^ The development of advanced LIBs is still going on in order to satisfy the growing demand of portable devices, electric vehicles (EV), and hybrid electric vehicles (HEV), which commonly require higher energy density and more durable cycle life.^1^, ^2^, ^3^ As the performance of LIBs heavily fluctuates with materials that they are composed of, intensive studies on key materials look essential for future advanced battery system.^4^, ^5^, ^6^ Bearing it in mind, the phosphate (PO_4_)^3−^ based materials are considered as a highly promising cathode candidate because these materials contain both mobile lithium cations and redox‐active metal sites shrouded within a rigid phosphate network.^6^, ^7^, ^8^ Especially, compared with lithium transition metal oxides, it displays remarkable electrochemical and thermal stability, as well as comparable energy density.^6^, ^7^

Among many phosphate‐based materials, monoclinic Li_3_V_2_(PO_4_)_3_ (LVP) has attracted much attention due to its excellent cycling stability, relatively high reaction potential (≈3.8 V vs. Li/Li^+^) and large theoretical capacity (197 mAh g^−1^ when all three lithium atoms are reversely extracted/inserted) with the abundance of vanadium resources in nature.^3^, ^9^, ^10^ In particular, its 3D structure framework composed of slightly distorted VO_6_ octahedra and PO_4_ tetrahedra sharing oxygen vertex provides large interstitial space that makes lithium ions capable of moving fast inside the structure.^11^, ^12^, ^13^ However, just like other metal phosphate group, it shows limited electronic conductivity (10^−8^–10^−9^ S cm^−1^), which is critically against the commercialization of LVP.^6^, ^14^, ^15^ Hence, its low electronic conductivity should be improved without sacrificing its significant advantage in terms of lithium ion diffusivity to utilize LVP as a commercial cathode material in the near‐future.

It is well known that the surface coating with electronically or ionically conductive materials can facilitate the movement of electrons or lithium ions inside active materials, finally realizing significantly enhanced electrochemical performances.^16^, ^17^, ^18^ Because of this reason, the strategic selection looks essential to choose the most proper coating materials to improve the electrochemical performances of electrode materials. Among various candidates for coating materials, carbon coating has been the most common method to encapsulate the surface of electrode materials because of its multifunctional properties such as high electronic conductivity, good conformality, and high stability. As a result, it has contributed to enhancing electronic conductivity, preventing transition metal dissolution, and suppressing volume expansion in electrode materials.^19^, ^20^, ^21^, ^22^ However, even though carbon is one of the most excellent electronic conductors among various reported coating materials, its intrinsic ionic conductivity is not good contrary to expectation.^23^ In fact, lithium ions can pass through the carbon layer just in case that the crystal defects exist in the corresponding carbon layer.^24^, ^25^, ^26^, ^27^ It means that from the viewpoint of lithium ion diffusion solely, bare surface looks more advantageous for lithium ion diffusion than the coated surface with carbon layer because it acts as another barrier that lithium ions should penetrate.^28^, ^29^ Despite this problem, carbon network is still necessary for active materials because it would help to raise electronic conductivity as well known. Thus, a sagacious design for the composite structure simultaneously enabling high electronic conductivity and ionic diffusivity is really required to reach the full potential performance of electrode materials.

Considering that lithium ion diffusion is quite restricted in the interface between electrolyte and electrode materials, and the resistance at electrolyte/electrode interface almost dominates the whole electrode resistance, we designed a rational composite structure composed of carbon fiber and active material particles to facilitate electron transport and lithium ion diffusion at the same time.^30^, ^31^ To realize the corresponding unique structure, we utilized a simple synthetic method based on electrospinning combined with controlled heat‐treatment. Finally, the unique composite material which contains nanosized active materials anchored in carbon nanofibers was successfully synthesized. Through the protruded surface of active material, not only lithium ion diffusion distance can be reduced but also direct contact between electrolyte and electrode material can be made, which is definitely favorable for lithium diffusion.^32^, ^33^, ^34^, ^35^, ^36^, ^37^, ^38^, ^39^, ^40^ Simultaneously, by constructing 1D carbon network, efficient electron transport into the active material can also be secured toward superb kinetics. In this study, we explored the method for realizing the unique structure, in which LVP particles are anchored in carbon nanofibers, based on electrospinning and controlled heat‐treatment. Also, it was observed how the formation and protrusion of LVP particles out of carbon fiber happens to figure out what is the most important parameter to realize this unique structure. Finally, the formation of this structure turned out to depend on typical nucleation‐growth behavior based on surface diffusion and Ostwald ripening. With different structures of LVP‐carbon composites, we could successfully demonstrate the importance of above‐mentioned unique composite structure in terms of electrochemical performances.

## 2 Results and Discussion

**Figure 1:**
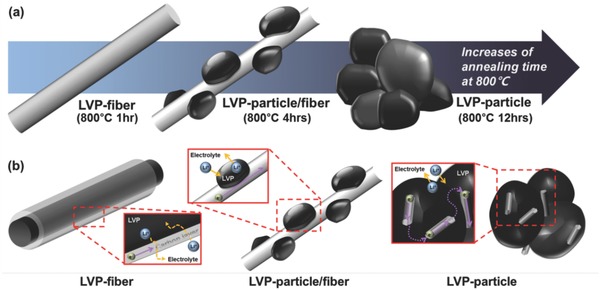
a) Schematic diagram for the morphological change of LVP/carbon nanofiber composites with heat‐treatment time at 800 °C, which show the protrusive growth of LVP particles out of carbon nanofibers and b) illustration for lithium ion and electron transport behaviors inside various LVP/carbon nanofiber composites including LVP‐fiber, LVP‐particle/fiber, and LVP‐particle.

**Figure** **1**a shows the schematic diagram for the growth mechanisms of LVP/carbon nanofibers with controlled heat‐treatments conditions. In here, LVP component and carbon nanofiber component are represented by black and light gray color, respectively. When we annealed LVP precursor fiber at 800 °C for 1 h, smooth‐surfaced fiber was only observed as indicated in the first part of Figure 1a. However, when the heat‐treatment time was increased up to 4 h, oval‐shaped LVP particles appeared on the surface of carbon nanofiber. When the heat‐treatment time is maintained for 12 h, carbon nanofiber almost disappeared and only LVP particles remained. Each sample maintained for 1, 4, and 12 h at 800 °C was each named as LVP‐fiber, LVP‐particle/fiber, and LVP‐particle, which well represents the structural characteristics of samples. When considering the way how the morphology of samples is changing during the heat‐treatment, it seems to predominantly affect the grain or particle growth by facilitating movement of atoms or molecules through the several mechanisms such as surface diffusion, evaporation–condensation, and lattice diffusion. The increase of heat‐treatment time at elevated temperature is also accompanied by the reduction of carbon nanofiber not only making its diameter thinner but also reducing V^5+^ to V^3+^. This series of process looks very important to regulate the peculiar structure of LVP/carbon nanofiber composite. Figure 1b illustrates lithium ion and electron movement behavior inside this composite structure. As aforementioned, LVP‐fiber is unfavorable for fast lithium ion diffusion due to the carbon layer that covers LVP parts. Actually, the presence of carbon layer suppresses or delays ion diffusion because it acts as an additional barrier which lithium ions should penetrate through. However, this uniform carbon layer for LVP‐fiber is beneficial in terms of electron transport. In comparison, LVP‐particle/fiber with exposed LVP surface can be much easily accessed by lithium ions due to the secured direct contact between LVP particles and electrolyte. Small size of LVP particles in LVP‐particle/fiber is regarded as another merit of this sample that can significantly shorten the distance for lithium ion diffusion and electron transport. Furthermore, LVP‐particle/fiber provides the interconnected electron pathways alongside 1D carbon nanofiber network. However, when the particle size of LVP particle is further increased, this carbon nanofiber network can be disconnected and finally disappear together with the growth of LVP particle as shown in LVP‐particle. The corresponding structure of LVP‐particle looks completely unfavorable for electron transport even if the contact area between LVP‐particle and electrolyte can be extended finally facilitating lithium ion diffusion a little more. More detailed relationship between structures and electrochemical properties in these LVP‐based samples will be discussed as follows.

**Figure 2:**
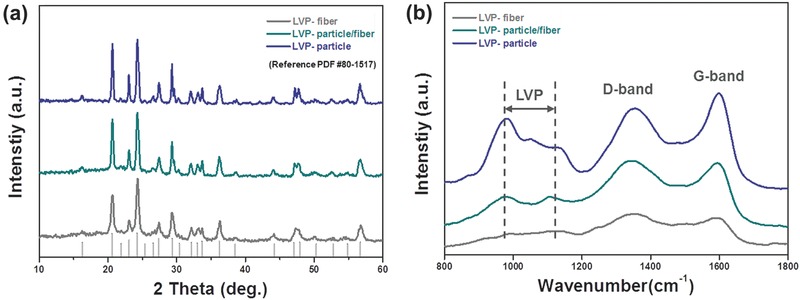
a) XRD patterns and b) Raman spectra of LVP/carbon nanofiber composites with different heat‐treatment time.

X‐ray diffraction (XRD) patterns of LVP samples with different heat‐treatment time are shown in **Figure** **2**a. All of the diffraction peaks are well matched with the reference peaks which come from the P2_1_/n space group of monoclinic LVP. As shown in Figure 2a, when the heat‐treatment time was increased, the crystallinity of LVP was gradually improved together with the morphological change from fiber to particle, which is demonstrated by the narrowed full width half maximum of peaks. In particular, LVP‐fiber showed notable additional shoulder peak around 2θ = 15°–35° region that are partially overlapped with monoclinic LVP peaks. Compared to LVP particle/fiber and LVP particle, larger amount of amorphous carbon inside LVP‐fiber demonstrated by this shoulder peak can result from relatively short heat‐treatment time that is just enough to allow amorphous carbon to be formed from citric acid and polyvinylpyrrolidone (PVP) residues. Naturally, the shoulder peak gets weaker with the extended heat‐treatment time resultantly indicating the decrease of amorphous carbon. This phenomenon can also be demonstrated from thermodynamic gravimetric analysis (TGA) as shown in Figure S1 (Supporting Information). Typically, the weight loss around 400–500 °C in TGA curve corresponds to the decomposition of carbon component implying that total carbon contents inside LVP‐based composite samples gradually decreased with the increment of heat‐treatment time. To investigate the nature of carbon inside the composite and compare the crystallinity of LVP phases, Raman spectra were obtained as shown in Figure 2b. All of spectra displayed two apparent peaks ascribed to D‐band near 1350 cm^−1^ and G‐band near 1600 cm^−1^, which each represents disordered carbon and crystalline carbon.^41^, ^42^, ^43^ Herein, as the heat‐treatment time was increased, the intensity ratio of D‐band to G‐band (*I*_D_/*I*_G_) were steadily decreased, which indicates the improved crystallinity of carbon inside this composite. Meanwhile, other peaks located in the range between 977 and 1106 cm^−1^ indicate the presence of monoclinic LVP crystal phase. Interestingly, the intensity for LVP crystal phase rises up as the heat‐treatment time increases. LVP‐fiber just showed weak and broad peak around this region, while much clear signal was detected in LVP‐particle/fiber and LVP‐particle.^44^, ^45^ Considering the characteristics of Raman analysis, this result seems to be in good agreement with TGA analysis results. Because the area irradiated by Raman laser is mainly restricted to the surface region of sample due to its low energy, Raman spectra tend to give the information around the surface of sample. Thus, the surged intensity ratio of LVP crystal compared to carbon species in LVP‐particle/fiber and LVP‐particle suggests that the exposure of LVP particles looks distinctive in not only LVP‐particle but also LVP‐particle/fiber because the carbon species are enough decomposed with the prolonged heat‐treatment time over 4 h. It was revealed that just through the variation of heat‐treatment time, the synergistic structure of LVP/carbon composite could be realized with the decrease of carbon contents as well as the surge of crystalline LVP contents. In order to figure out the effect of carbon on the electronic conductivity of LVP/carbon composites, four‐wire resistor measurements were conducted, which obtains the electrical resistance inversely proportional to the electronic conductivity as indicated in Figure S2 (Supporting Information).^46^ Typically, four‐wire resistor measures the electrical resistance of sample by detecting the voltage change with the current. The calculated values of electrical resistance are summarized in Table S1 (Supporting Information). It can be noted here that the electrical resistance of LVP/carbon composite was also increased as the heat‐treatment time was extended. Although the carbon contents in the samples decreased with the increment of heat‐treatment time, the presence of carbon in LVP samples looks clearly beneficial to improve its electronic conductivity.

**Figure 3:**
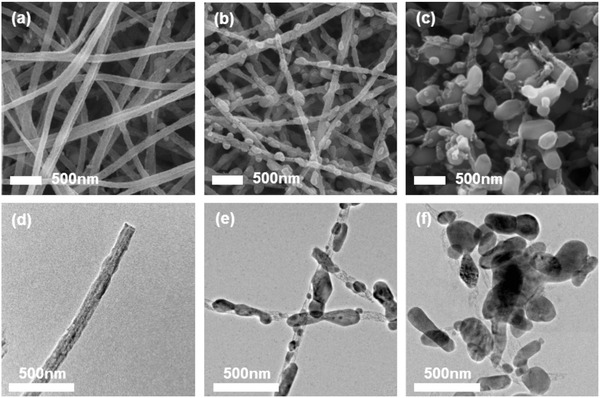
SEM images of LVP/carbon nanofiber composites with different heat‐treatment time. a) LVP‐fiber, b) LVP‐particle/fiber, and c) LVP‐particle, and TEM images of d) LVP‐fiber, e) LVP‐particle/fiber, and (f) LVP‐particle.

Morphological changes of LVP/carbon composites with different heat‐treatment time were investigated with scanning electron microscope (SEM) (**Figure** **3**a–c) and transmission electron microscope (TEM) analyses (Figure 3d–f). Significant changes in the particle size of LVP and the morphology of carbon nanofiber were observed as the heat‐treatment time increased. For LVP‐fiber sample (Figure 3a,d), it shows smooth‐surfaced fibrous structure without any distinct characteristics, which is similar to that of as‐electrospun nanofibers before any heat‐treatment in Figure S3a (Supporting Information). However, LVP/carbon composite underwent an interesting gradual change as heat‐treatment time increased. When heat‐treatment time was over 1 h, LVP particles in the composite began to grow up or agglomerate as shown in Figure S3b (Supporting Information) rendering oval particles to be anchored in carbon nanofibers. Then, the size of LVP particles became bigger while the thickness of carbon nanofibers became thinner. When heat‐treatment time reached 4 h (LVP‐particle/fiber), it showed protruded LVP particles and carbon nanofibers as shown in Figure 3b,e. This peculiar morphological change involving the evolution and growth of ceramic particles from the mixture of polymer and ceramic precursors seems to depend on not only the initial sintering process like evaporation–condensation or surface diffusion but also typical Ostwald ripening, which accompanies the nucleation of LVP crystal and the following selective growth of larger nuclei or grain with positive curvature. When this sequential crystal growth process was maintained until 8 h (Figure S3c, Supporting Information), LVP particles grew up much bigger and when it was maintained until 12 h, LVP particles with larger particle size and inhomogeneous size distribution were generated as shown in Figure 3c,f. Here, carbon nanofibers almost disappeared probably due to the continuous carbothermal reaction during heat‐treatment in good agreement with TGA and Raman analyses data. Actually, a gradual weight loss of carbon species was observed with the growth of LVP particles, finally making LVP particles protrude out of carbon nanofibers. After LVP particles became enough large and exposed to be irradiated by Raman laser, its crystallinity looked more exaggerated in Raman spectra (Figure 2a), but we could make sure that LVP‐fiber after 1 h heat‐treatment has almost same crystallinity as LVP‐particle/fiber and LVP‐particle through XRD analyses (Figure 2b). Based on these comprehensive results, we could suggest the formation mechanism for this peculiar LVP/carbon composite as indicated in Figure 1. Even though the extended heat‐treatment led to the growth of LVP particle, the particle size of LVP/carbon composites obtained by electrospinning process was a lot smaller than that by conventional process without electrospinning (Figure S3d, Supporting Information). In order to more clearly see the structure of LVP/carbon composite, TEM images in higher resolution were obtained and incorporated as Figures S4 and S5 (Supporting Information). The thickness of carbon layer on the surface of LVP‐fiber was about 15.09 nm. In LVP‐particle/fiber and LVP‐particle sample, protruded LVP particles can be clearly discriminated from carbon parts even from high‐resolution TEM (HR‐TEM) images (Figure S5, Supporting Information). Distinct lattice fringes were found in the LVP particle, whose width was 4.21 and 4.29 Å corresponding to (0 2 0) planes of LVP phase. As indicated in Figure S6 (Supporting Information), average particle size of LVP‐particle/fiber and LVP‐particle is 114.06 and 304.89 nm each, and the particle size distribution of LVP‐particle/fiber is much more homogeneous compared to that of LVP‐particle. To confirm the structure of LVP‐particle/fiber, elemental mapping images are included in Figure S7 (Supporting Information). Considering that carbon is the best electronic conductor but is not good ionic conductor, LVP particles anchored in carbon nanofibers (LVP‐particle/fiber) can be one of the best composite structures, which are able to get over the clear limitation of typical carbon coating strategy by simultaneously facilitating not only electron transport but also ionic transports.

**Figure 4:**
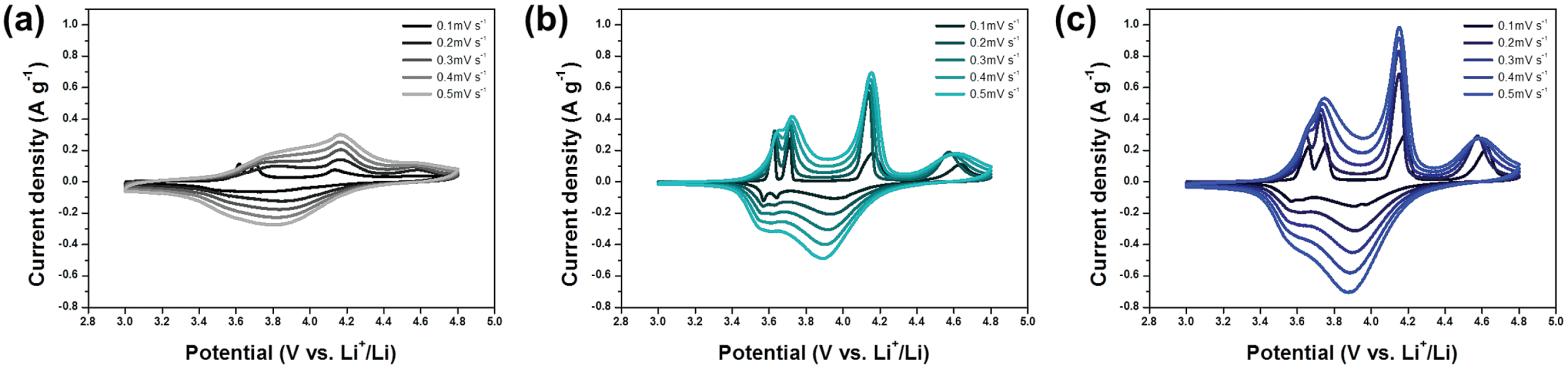
Cyclic voltammetry curves of LVP/carbon nanofiber composites. a) LVP‐fiber, b) LVP‐particle/fiber, and d) LVP‐particle obtained at different scan rates ranging from 0.1 to 0.5 mV s^−1^.

Cyclic voltammetry (CV) curves of LVP/carbon composites obtained between 3.0 and 4.8 V with the variation for scan rate of 0.1–0.5 mV s^−1^ are shown in **Figure** **4**. During lithium extraction/insertion, LVP‐based materials normally show four oxidation peaks during charge and three reduction peaks during the subsequent discharge in the voltage range of 3.0–4.8 V (vs. Li^+^/Li), which are related to a series of phase transitions of crystalline LVP.^7^, ^10^, ^47^, ^48^ The whole electrochemical reactions are explained as follows

(1) $${\mathrm{Li}}_{3}V_{2}{\left({\mathrm{PO}}_{4}\right)}_{3}-0.{\mathrm{5Li}}^{+}-0.{\mathrm{5e}}^{-}↔{\mathrm{Li}}_{2.5}V_{2}{\left({\mathrm{PO}}_{4}\right)}_{3}$$

(2) $${\mathrm{Li}}_{2.5}V_{2}{\left({\mathrm{PO}}_{4}\right)}_{3}-0.{\mathrm{5Li}}^{+}-0.{\mathrm{5e}}^{-}↔{\mathrm{Li}}_{2}V_{2}{\left({\mathrm{PO}}_{4}\right)}_{3}$$

(3) $${\mathrm{Li}}_{2}V_{2}{\left({\mathrm{PO}}_{4}\right)}_{3}-1.{\mathrm{0Li}}^{+}-1.{\mathrm{0e}}^{-}↔{\mathrm{LiV}}_{2}{\left({\mathrm{PO}}_{4}\right)}_{3}$$

(4) $${\mathrm{LiV}}_{2}{\left({\mathrm{PO}}_{4}\right)}_{3}-1.{\mathrm{0Li}}^{+}-1.{\mathrm{0e}}^{-}↔V_{2}{\left({\mathrm{PO}}_{4}\right)}_{3}$$

Two oxidation peaks at 3.62 and 3.71 V during charge can be assigned to the extraction of first lithium ion with the oxidation of vanadium from V^3+^ to V^3+^/V^4+^, which corresponds to reactions (1) and (2). Another oxidation peak at 4.16 V results from the extraction of second lithium ion with the complete vanadium oxidation from V^3+^/V^4+^ to V^4+^ in reaction (3). Finally, the oxidation peak around 4.63 V represents the extraction of last lithium ion with the vanadium oxidation from V^4+^ to V^5+^ as indicated in reaction (4).^10^, ^47^, ^48^, ^50^ The following discharge has been regarded as kinetically difficult process due to the limited ionic/electronic conductivity of V_2_(PO_4_)_3_ framework.^49^, ^50^ During subsequent discharge, broad reduction peak at 3.95 V would be observed, which originated from the initial reinsertion of lithium ion into V_2_(PO_4_)_3_, which is a kind of solid solution reaction. Another two reduction peaks appear near 3.64 and 3.57 V, which are attributed to the last lithium ion insertion.^10^, ^47^, ^48^ Compared to LVP‐particle/fiber and LVP‐particle, LVP‐fiber (Figure 4a) shows somewhat smooth peaks with lower intensity. As the scan rate increased gradually, the peak intensity for LVP‐fiber a little rose up in accordance with typical feature of CV curves, but the difference between oxidation and reduction potentials became larger and the peak during discharge is blurred indicating that its electrochemical reaction is quasi‐reversible and kinetically slow even though LVP‐fiber showed the highest electronic conductivity thanks to high contents of carbon species.^51^, ^52^ Meanwhile, LVP‐particle/fiber (Figure 4b) and LVP‐particle (Figure 4c) showed more distinct oxidation or reduction peaks, which may come from the typical electrochemical reaction of monoclinic LVP phase. Some reduction peaks in LVP‐particle was overlapped with each other between 3.6 and 3.8 V demonstrating its irreversibility when the scan rate was increased, while LVP‐particle/fiber almost maintained its distinctive peaks even at high scan rates. Similar phenomenon was also observed during charge (oxidation). Two oxidation peaks around 3.5–3.6 V can be clearly discriminated even at high rates in CV curves of LVP‐particle/fiber, but these peaks are overlapped in the CV curves of LVP‐fiber and LVP‐particle. Because the peak overlapping in CV curves generally happens when ions cannot be completely extracted or inserted in electrode material, the apparent peak separation in LVP‐particle/fiber looks like proving that this composite structure is kinetically favorable enough to allow fast charge/discharge.

**Figure 5:**
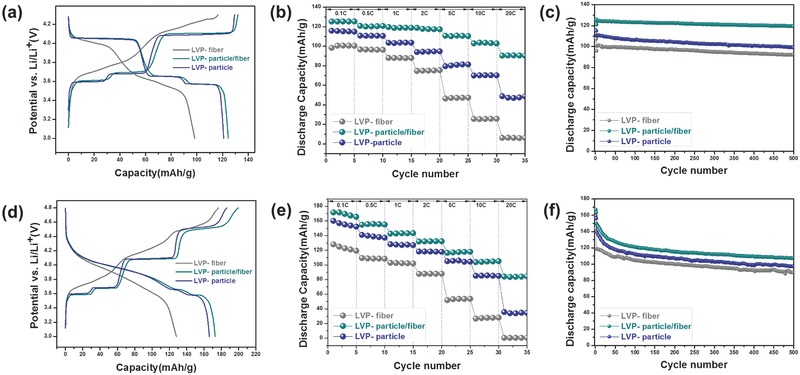
Galvanostatic charge–discharge curves of LVP/carbon nanofiber composites in two different voltage ranges a) 3.0–4.3 V and d) 3.0–4.8 V. Rate capability results of LVP/carbon nanofiber composites with the variation of C‐rate from 0.1 to 20 C in b) 3.0–4.3 V and e) 3.0–4.8 V. Cycling stabilities of LVP/carbon nanofiber composites in c) 3.0–4.3 V and f) 3.0–4.8 V (C‐rate was 0.1 C for first two cycles, and 1 C was applied for the following 500 cycles).

**Figure** **5**a,d shows galvanostatic charge–discharge curves of LVP/carbon composites obtained either between 3.0 and 4.3 V or between 3.0 and 4.8 V. By limiting the charge voltage to 4.3 V, which is the median voltage between the theoretical voltage for second lithium ion extraction (4.16 V) and that for third lithium ion extraction (4.63 V), we could adjust the number of lithium ions participating in the corresponding electrochemical reaction below two.^10^, ^51^ The charge–discharge reactions of LVP/carbon composites are generally based on several consecutive phase transitions as indicated by voltage plateaus. During the initial charge, the first lithium ion was extracted through two oxidation reactions at 3.60 and 3.68 V, whereas the second lithium ion tended to be fully extracted through the single potential plateau at 4.09 V. Subsequent discharge looks quite reversible based on the discharge profiles. The voltage plateaus that correspond to lithium ion extraction and insertion are well matched with the CV results. When the charging voltage was increased up to 4.8 V, the last third lithium ion also came to participate in the oxidation that leads to a plateau at 4.53 V (Figure 5d). During the following discharge, a solid‐solution behavior featured by S‐shape curve was found for all LVP samples until two lithium ions were completely inserted into V_2_(PO_4_)_3_ framework.^7^, ^53^, ^54^, ^55^ The morphological or microstructural effect on different of LVP/carbon composites was demonstrated through their electrochemical tests. LVP‐fiber, LVP‐particle/fiber, and LVP‐particle showed the initial discharge capacities each coming up to 98.37 mAh g^−1^, 123.44 mAh g^−1^, and 120.87 mAh g^−1^ between 3.0 and 4.3 V. However, when the charging voltage was extended to 4.8 V, LVP‐fiber, LVP‐particle/fiber, and LVP‐particle delivered the enhanced capacities of 128.11, 172.93, and 166.16 mAh g^−1^, respectively. As discussed earlier, the participation of the third lithium ion in electrochemical reaction is not kinetically favored. Thus, the highest capacity of LVP‐particle/fiber among LVP/carbon composites implies that its morphological or microstructural characteristics are really advantageous. To further compare the structural advantages of LVP/carbon composites, rate capability test was conducted by varying C‐rates from 0.1 to 20 C (to calculate C‐rates, the theoretical capacity between 3.0 and 4.3 V was assumed as 131 mAh g^−1^, while 197 mAh g^−1^ was the theoretical capacity for the voltage range between 3.0 and 4.8 V) as shown in Figure 5b,e. The LVP‐particle/fiber showed not only highest capacity reaching 126 mAh g^−1^ (3.0–4.3 V) and 172 mAh g^−1^ (3.0–4.8 V) at 0.1 C, but also displayed the best capacity retention at 20 C in comparison with LVP‐fiber and LVP‐particle. The discharge capacities of LVP‐particle/fiber at 20 C were 72.09% (3.0–4.3 V) and 48.96% (3.0–4.8 V) of those at 0.1 C, whereas the discharge capacities of LVP‐fiber and LVP‐particle were 6.23% and 42.10% compared to those at 0.1 C with the charging voltage of 4.3 V cut, and 0.28% and 22.27% with the increased charging voltage of 4.8 V. The cyclic stabilities of all samples were obtained at 1 C as shown in Figure 5c,f. Herein, LVP‐particle/fiber exhibited the best cycling stability for 500 cycles. When the charging voltage was restricted to 4.3 V (Figure 5c), LVP‐particle/fiber delivered a reversible capacity of 119.55 mAh g^−1^ after 500 cycles, which maintained 98.73% of its initial discharge capacity, while the discharge capacities of LVP‐fiber and LVP‐particle were just 92.41 mAh g^−1^ and 99.36 mAh g^−1^, respectively. When the charging voltage was increased to 4.8 V (Figure 5f), LVP‐particle/fiber still retained the highest capacity of 107.69 mAh g^−1^ corresponding to 71.83% of the initial capacity, but LVP‐fiber and LVP‐particle each showed 89.85 and 97.26 mAh g^−1^ after 500 cycles. Charge–discharge profiles during rate‐capability test and cycling test are shown in Figure S8 (Supporting Information). SEM images before or after cycles are included in Figure S9 (Supporting Information). As indicated in the images, even after 500 cycles, the morphology of LVP fibers and particles shows scarce change probably thanks to the good structural stability of LVP. Actually, due to the covalently bonded oxygen atoms inside the LVP material, LVP has been reported to be structurally stable even when lithium ions are extracted from itself.^7^

**Figure 6:**
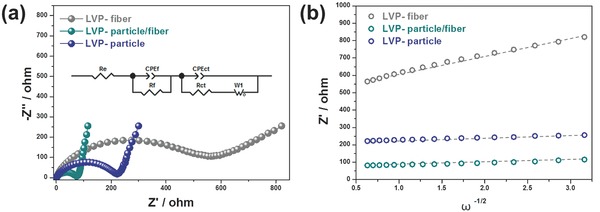
a) EIS spectra of LVP/carbon nanofiber composites after 2 cycles (3.0–4.8 V). Inset of (a) is equivalent circuit corresponding to the impedance spectra. b) Relationship between the *Z*′ and ω^−1/2^ in the low frequency region of LVP/Carbon composites.

In order to investigate the kinetic properties of LVP/carbon composites, electrochemical impedance spectroscopy (EIS) measurement was conducted. Before the measurement, electrodes were galvanostatically charged and discharged for two cycles between 3.0 and 4.8 V at low rate of 0.1 C in order to assure the penetration of electrolyte into electrode, structural change, and the formation of stable solid electrolyte interface layer on the surface of active material.^56^ The Nyquist plot (**Figure** **6**a) consisted of two parts, a depressed semicircle in high‐frequency region and an inclined straight line in low‐frequency region. From the semicircle in high‐frequency region, charge‐transfer resistance (*R*_ct_) could be deduced, which corresponds to the resistance between electrolyte and LVP electrode. From the inclined line in low‐frequency region, lithium ion diffusivity could be calculated using a certain formula.^23^, ^49^ From the fitting results, *R*_ct_ of LVP‐particle/fiber corresponded to 68.72 Ω, which was much smaller than those of LVP‐fiber (489.50 Ω) and LVP‐particle (88.82 Ω). This result indicates that lithium ion transfer is much easier not only inside LVP‐particle/fiber electrode but also between the electrode and electrolyte. (The detailed values of resistance that could be obtained from fitting result are summarized in Table S2, Supporting Information) On the other hand, lithium diffusion coefficients of LVP samples were calculated from the inclined line in low‐frequency region by the following equation^57^, ^58^

(5) $$D_{\mathrm{Li+}}=R^{2}T^{2}\mathrm{/2}A^{2}n^{4}F^{4}C^{2}\delta^{2}$$

In this equation, *R* is gas constant (8.3143 J mol^−1^ K^−1^), *T* is absolute temperature (298 K at room temperature), *A* is the surface area of electrode (1.13 cm^2^), *n* is the number of electrons per molecule during oxidation (*n* = 1), *F* is faraday constant (96485.3365 C mol^−1^), *C* is the concentration of lithium ions in the corresponding electrode material (3.7 × 10^−3^ mol cm^−3^), and δ is Warburg coefficient which can be calculated from the correlation between frequency and impedance, according to the following formula^57^, ^58^

(6) $$Z^{\prime }=R_{e}+R_{\mathrm{ct}}+\delta \omega^{-1/2}$$

The ω is frequency in the low frequency region and from the obtained δ value, lithium ion diffusivity could be calculated. As a result of calculation, Warburg coefficient and lithium ion diffusivity of LVP samples are shown in **Table** **1**. From this result, LVP‐particle/fiber sample demonstrated its highest lithium ion diffusivity followed by LVP‐particle and LVP‐fiber in sequence, which well agrees with the electrochemical test results in Figure 5. The superior lithium ion diffusion in LVP‐particle/fiber can be explained by the protrusion of LVP particles out of carbon nanofiber, which renders lithium ions to directly go into the lattice structure of LVP from electrolyte, and in addition the small particle size of LVP can substantially shorten lithium ion transport distance while electron pathway is apparently secured alongside carbon nanofibers.

**Table 1:** Warburg coefficient (δ) and calculated lithium ion diffusivity (*D*_Li+_) from EIS measurement

Samples	δ (Ω cm^2^ s^−1/2^)	*D*_Li+_ (cm^2^ s^−1^)
LVP‐fiber	103.590	1.888 × 10^−13^
LVP‐particle/fiber	12.468	1.316 × 10^−11^
LVP‐particle	13.784	1.066 × 10^−11^

Based on the discussion above, we can conclude that high lithium ion diffusivity and superb electrochemical performances of LVP‐particle/fiber is associated with its special microstructural feature. As suggested in Figures 1 and 3, LVP‐particle/fiber has a unique microstructure which consists of carbon nanofibers and small LVP particles protruded out of carbon nanofibers. This structure could ensure not only high lithium ion diffusivity provided by the exposed particle surface region but also fast electron transport secured alongside the connected carbon nanofiber network. Meanwhile, other LVP samples such as LVP‐fiber and LVP‐particle had clear disadvantages in either lithium ion diffusivity or electronic conductivity finally delivering inferior electrochemical properties compared to LVP‐particle/fiber. Even if carbon coating technique has been regarded as the most effective way to improve the kinetics of electrode materials, this results made sure that the carbon layer on surface can interfere with the direct lithium ion transfer from electrolyte to electrode material. More importantly, the distinctive morphology or microstructure of LVP‐particle/fiber demonstrated how lithium ion diffusivity and electron transfer can be simultaneously maximized.

## 3 Conclusion

In summary, we successfully designed and realized LVP/carbon nanofiber comprised of LVP particles anchored in carbon nanofibers to simultaneously increase electronic conductivity as well as lithium ion diffusivity. Herein, the morphology or microstructure of LVP/carbon nanofiber composites was well modulated according to the typical sintering mechanisms at initial stage site such as surface diffusion, evaporation–condensation, etc. Interestingly, the apparent changes for LVP particle size and carbon nanofiber thickness were observed with heat‐treatment time finally giving the clue for the way how the unique LVP/carbon nanofiber structure is formed. By adjusting synthetic condition, we could grow up small LVP particles on carbon nanofibers in which some part of particle was protruded out of nanofiber. The unique microstructural features provided the chance to simultaneously secure fast lithium ion diffusion and electron transfer, which are essential for the enhanced electrochemical properties of electrode materials. Thanks to the synergistic effect of regulated LVP particle and carbon nanofiber, LVP/carbon fiber composite shows superb electrochemical performances. Actually, the optimized LVP/carbon fiber composite delivers high discharge capacities of 126 mAh g^−1^ (3.0–4.3 V) and 172 mAh g^−1^ (3.0–4.8 V) at 0.1 C, an impressive cyclic retentions (98.73% of the initial capacity was maintained after 500 cycles between 3.0 and 4.3 V) and a superb rate capability (the capacity at 20 C corresponded to 72.09% of that at 0.1 C between 3.0 and 4.3 V). Therefore, the unique structure reported here looks very promising to improve the kinetic properties of electrode materials. We demonstrated that the size and crystallinity of protruded LVP particles as well as the thickness and crystallinity of carbon nanofiber are very important physical parameters dominating the electrochemical properties of the corresponding composite cathode. This optimized unique structure can provide not only good electron pathways through 1D carbon nanofiber network but also showed good lithium ion diffusivity through the direct contact between electrolyte and LVP particles. Finally, this inventive idea can be considered as one of the most creative ones to not only overcome the limitation of conventional carbon‐coating strategies in terms of ionic diffusivity but also provide a clear solution for enhancing the kinetic properties of oxide‐based electrode materials including LVP.

## 4 Experimental Section

*Sample Preparation*: LVP samples were prepared by electrospinning method. First, stoichiometric amount of NH_4_VO_3_, NH_4_H_2_PO_4_, and CH_3_COOLi·2H_2_O were mixed in 5 mL of 14 wt% citric acid solution and magnetically stirred for 4 h in 60 °C oil bath. In here, citric acid was employed as a chelating agent and carbon source. After that, the mixed precursor was dropped slowly into the mixture solution of PVP 1.2 g (*M*_w_: 1,300,000) and 4.0 g deionized water and stirred for another 4 h. The mixed solution was loaded into a plastic syringe equipped with a 27‐gauge plastic needle. The needle was then connected to a high‐voltage power supply that generates DC voltage of 22.0 kV. The precursor solution was fed in the rate of 0.2 mL h^−1^ with syringe pump, and the distance between needle point and aluminum collector was 10 cm. The electrospun fibers were annealed at 800 °C maintaining for 1, 4, and 12 h at ramp rate of 2 °C min^−1^.

*Physical Characterization*: In order to characterize the crystal structure of LVP samples, X‐ray diffractometer (Rigaku Ultima IV) with Cu Kα radiation was used in the 2θ range of 10°–60°. The morphological structure and microstructure of the composite were obtained using field‐emission scanning electron microscopy (JEOL JSM‐6700F), and detailed nanostructure features were characterized by HR‐TEM (JEOL JEM‐3010) installed at the National Center for Inter‐university Research Facilities at Seoul National University. In order to see the carbon content of samples, TGA (Perkin Elmer, STA6000) was conducted in ambient air at a heating rate of 10 °C min^−1^ and carbon characteristics were measured with Raman spectroscopy (NRS‐3100) installed at the Hanyang Center for Research Facilities (Seoul). CV measurements were tested between 3.0 and 4.8 V in the scan rate of 0.1–0.5 mV s^−1^, and four‐wire resistor was conducted using (Keithley 4200 SCS) with making LVP samples in round‐shaped pellet with the thickness of around 0.5 mm. EIS measurement was conducted after first two cycles at frequency range from 1000 kHz to 100 mHz with sinusoidal voltage signal of 5 mV in amplitude as the perturbation at room temperature.

*Electrochemical Measurements*: The electrodes were prepared by mixing active material, acetylene black, and poly‐vinylidene fluoride in a weight ratio of 80:10:10 in *N*‐methyl pyrrolidone. The resulting slurry was pasted on aluminum foil current collector and dried at 120 °C for 5 h in a vacuum oven. After that, the electrodes were pressed and punched into circle shape in diameter of 1.3 cm. In here, the loading of the active material is about ≈2 mg cm^−2^. Electrochemical tests were performed using 2032 coin‐type cells with lithium metal as anode material and LiPF_6_ in 1:1 EC/DEC as electrolyte. Charge and discharge properties were examined at room temperature using WonAtech battery cycler in the voltage range of 3.0–4.8 V and 3.0–4.3 V (vs. Li^+^/Li).

## Supporting information

Supplementary
