# Supplementary material for: Carbon Nanofibers Heavy Laden with Li3V2(PO4)3 Particles Featuring Superb Kinetics for High‐Power Lithium Ion Battery
Source: Adv Sci (Weinh). 2017 May 12;4(9):1700128. doi: 10.1002/advs.201700128 (PMC5604389; doi:10.1002/advs.201700128)
Supplement: Supplementary file 1 — Supplementary [file ADVS-4-na-s001.pdf]

Copyright WILEY-VCH Verlag GmbH & Co. KGaA, 69469 Weinheim, Germany, 2017.

ADVANCED SCIENCE
Open Access

Supporting Information

for *Adv. Sci.,* DOI: 10.1002/advs.201700128

Carbon Nanofibers Heavy Laden with $Li_3V_2(PO_4)_3$ Particles Featuring Superb Kinetics for High-Power Lithium Ion Battery

*Jeongyim Shin, Junghoon Yang, Chernov Sergey, Min-Sang Song, and Yong-Mook Kang**

Copyright WILEY-VCH Verlag GmbH & Co. KGaA, 69469 Weinheim, Germany, 2016.

Supporting Information

Carbon nanofibers Heavy Laden with $Li_3V_2(PO_4)_3$ Particles Featuring Superb Kinetics for High-Power Lithium Ion Battery

*Jeongyim Shin, Junghoon Yang, Chernov Sergey, Min-Sang Song, Yong-Mook Kang**

J. Shin, J. Yang, Dr. C. Sergey, Prof. Y. –M. Kang
Department of Energy and Materials Engineering, Dongguk University, Seoul 100-715, Republic of Korea
E-mail: dake1234@dongguk.edu

Dr. M. –S. Song
Energy Material Lab, Material Research Center, Samsung Advanced Institute of Technology
Samsung Electronics, 130 Samsung-ro, Yeongtong-gu, Suwon-si, Gyeonggi-do, 16678, Republic of Korea

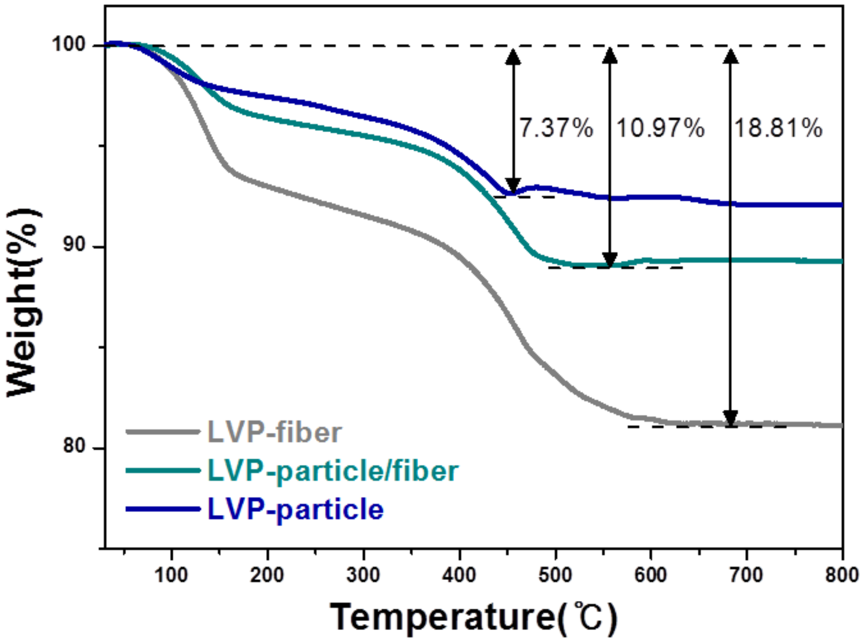

Figure S1. Thermodynamic gravimetric analysis (TGA) results of LVP/carbon nanofiber composites.

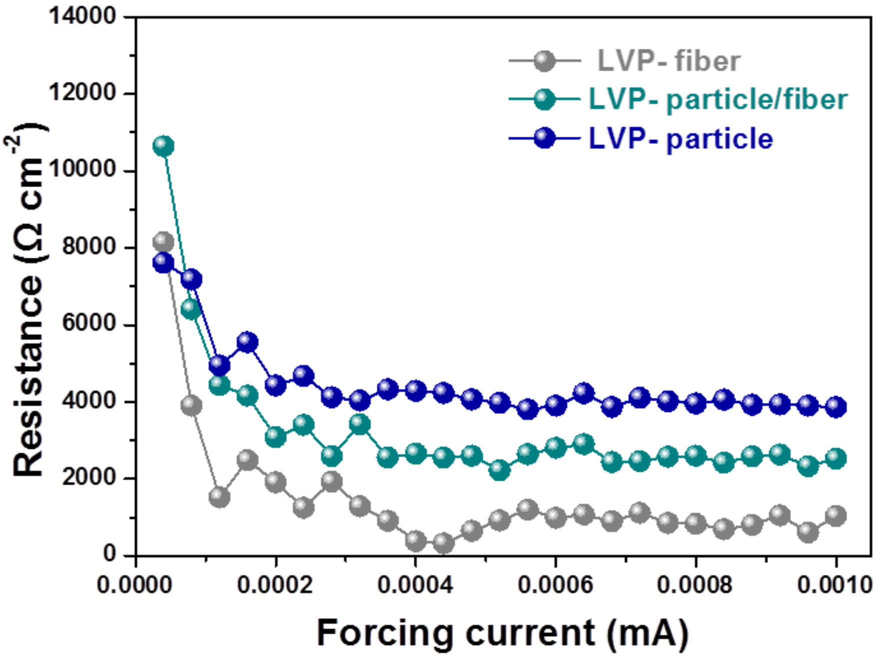

Figure S2. 4-wire resistor results of (a) LVP-fiber, (b) LVP-particle/fiber, and (c) LVP-particle.

Sample	Resistance (Ω cm^{-2})
**LVP- fiber**	893.3456
**LVP- particle/fiber**	2543.3896
**LVP- particle**	3983.1281

Table S1. Calculated resistances of LVP/carbon nanofiber composites from 4-wire resistor results.

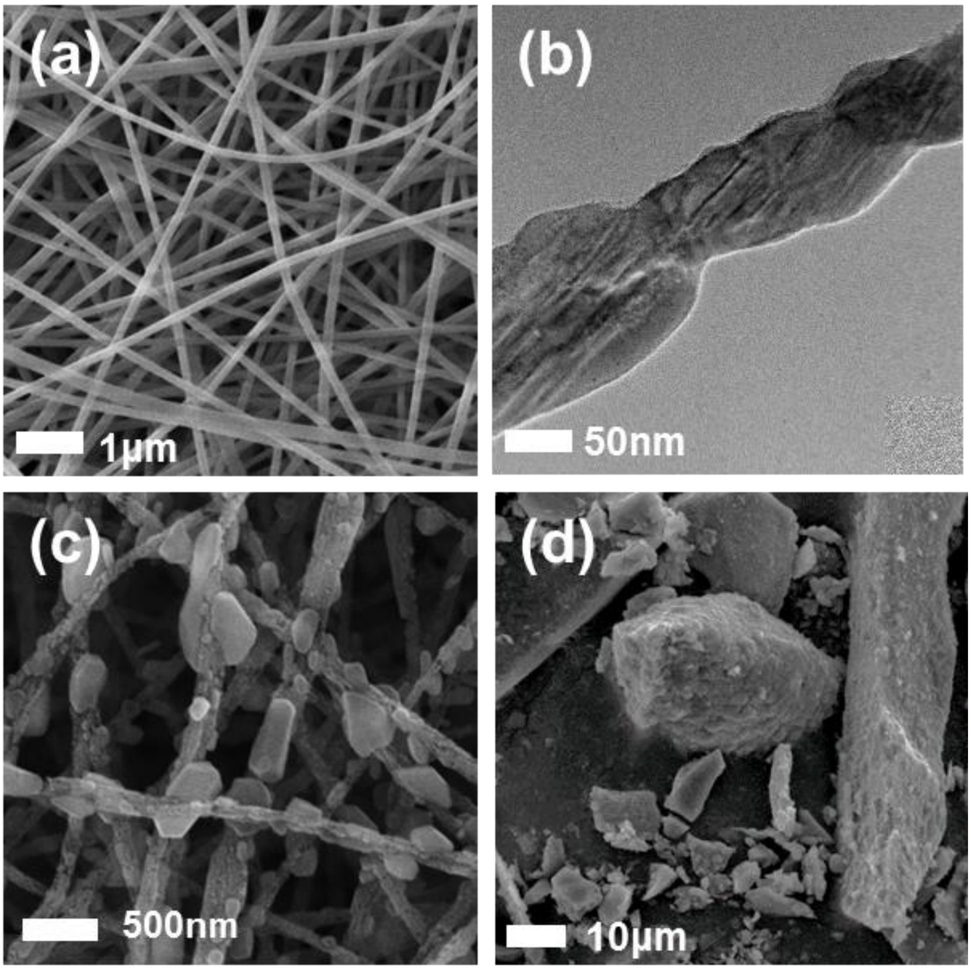

Figure S3. (a) SEM image of electrospun LVP precursors. (b) TEM image of LVP/carbon nanofiber composite annealed at 800°C for 2hrs. (c) SEM images of LVP/carbon nanofiber composite annealed at 800°C for 8hrs. (d) SEM image of LVP synthesized by conventional process without electrospinning.

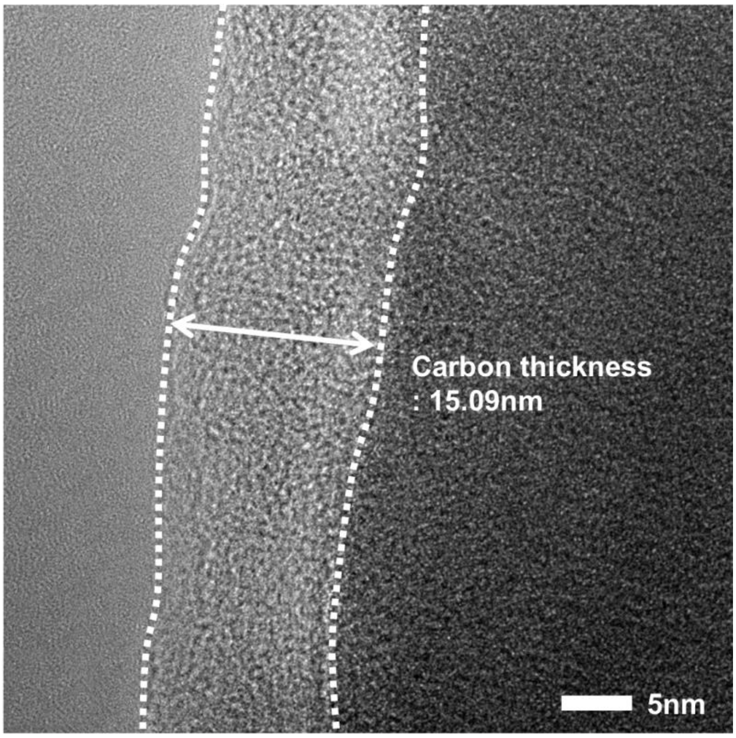

Figure S4. HR-TEM images of LVP-fiber showing the carbon layer on the surface.

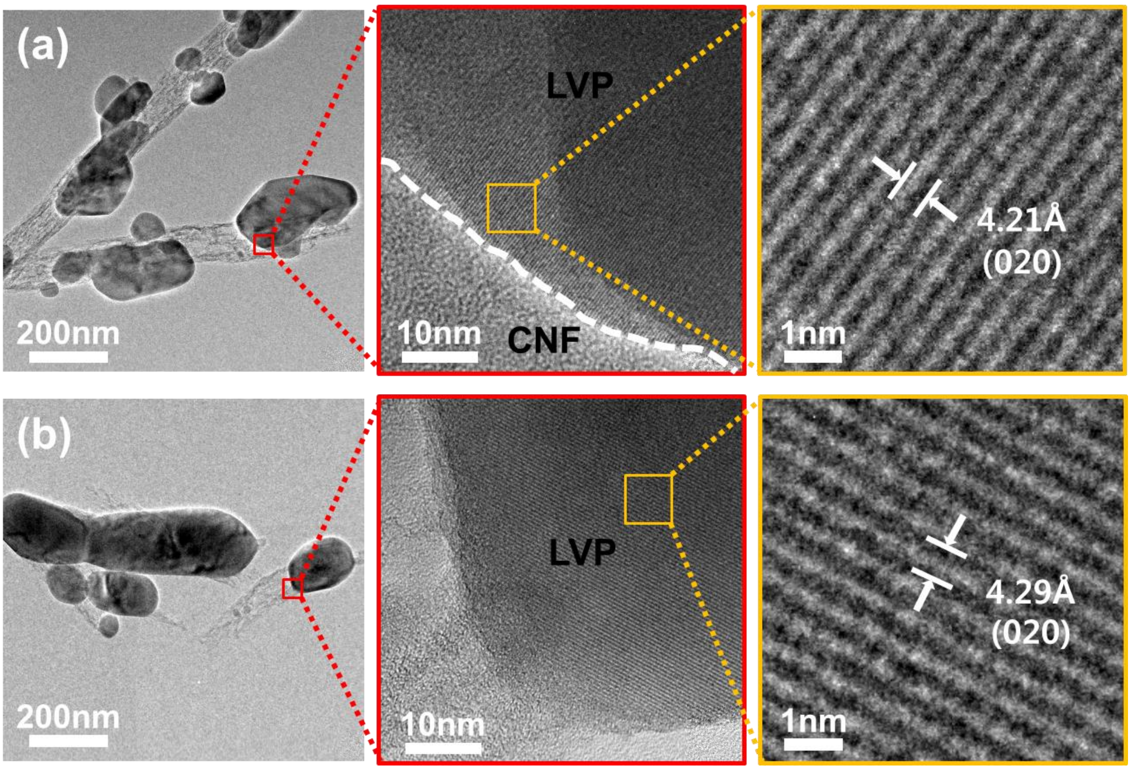

Figure S5. TEM images and HR-TEM images of (a) LVP-particle/fiber and (b) LVP-particle showing more precise structure and lattice fringe of crystalline LVP.

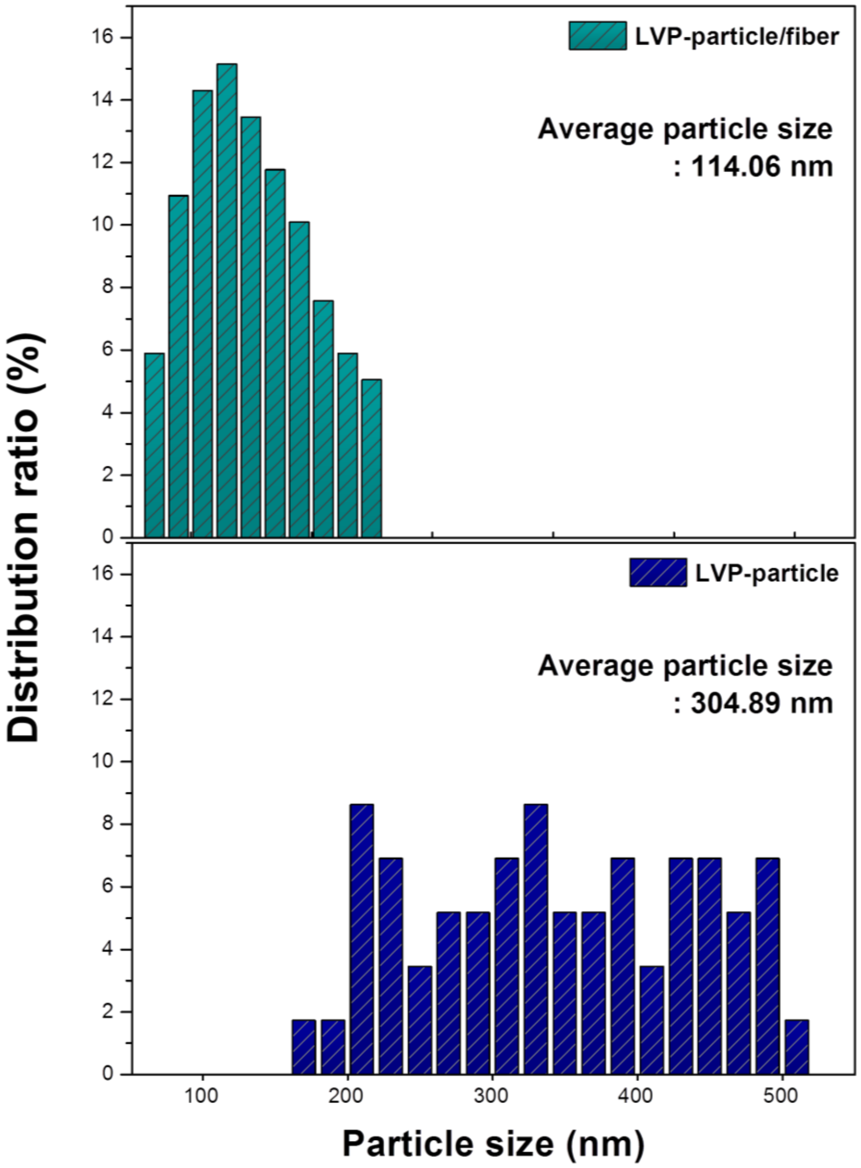

Figure S6. Particle size distribution of LVP-particle/fiber and LVP-particle samples.

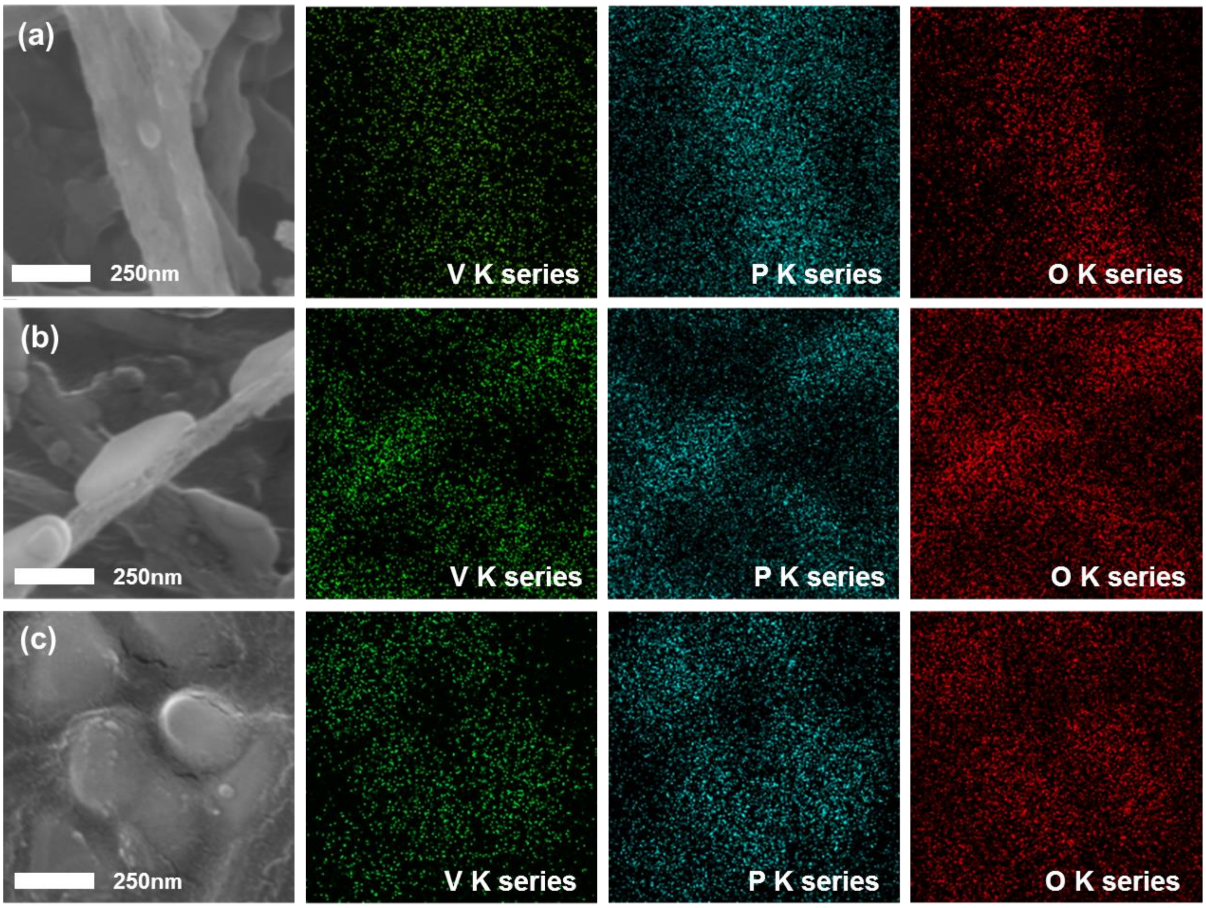

Figure S7. Elemental mapping images of (a) LVP-fiber, (b) LVP-particle/fiber, and (c) LVP-particle.

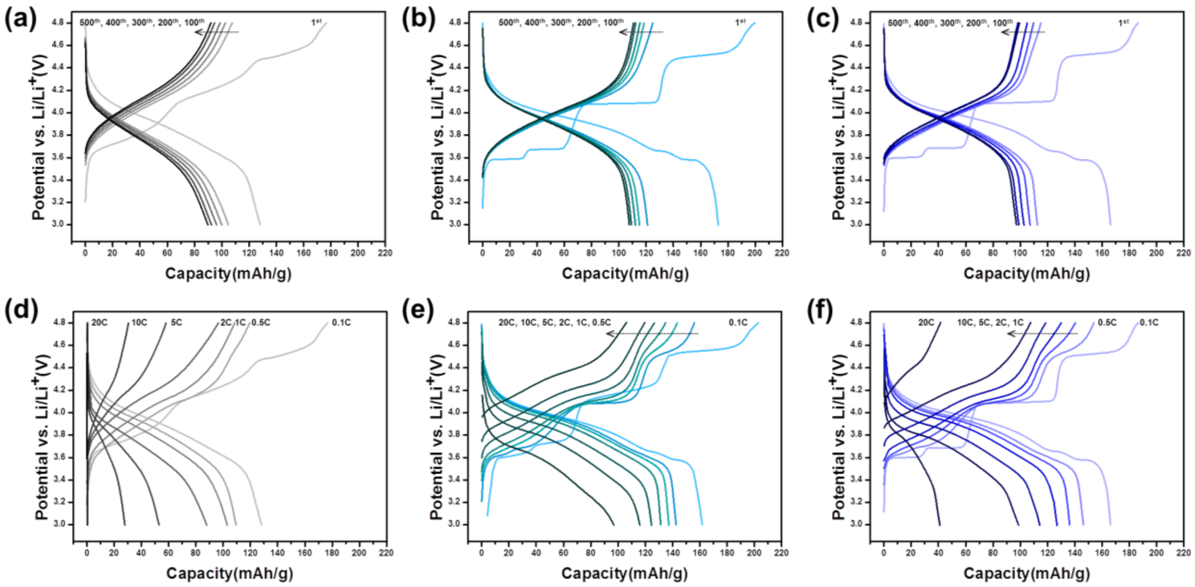

Figure S8. Galvanostatic charge-discharge curves of (a) LVP-fiber, (b) LVP-particle/fiber, and (c) LVP-particle during long cycles in 3.0 – 4.8 V (0.1 C for two cycles, and 1 C was applied for the following 500 cycles). Galvanostatic charge-discharge curves of (d) LVP-fiber, (e) LVP-particle/fiber, (f) LVP-particle during rate capability test from 0.1 to 20 C in 3.0 – 4.8 V.

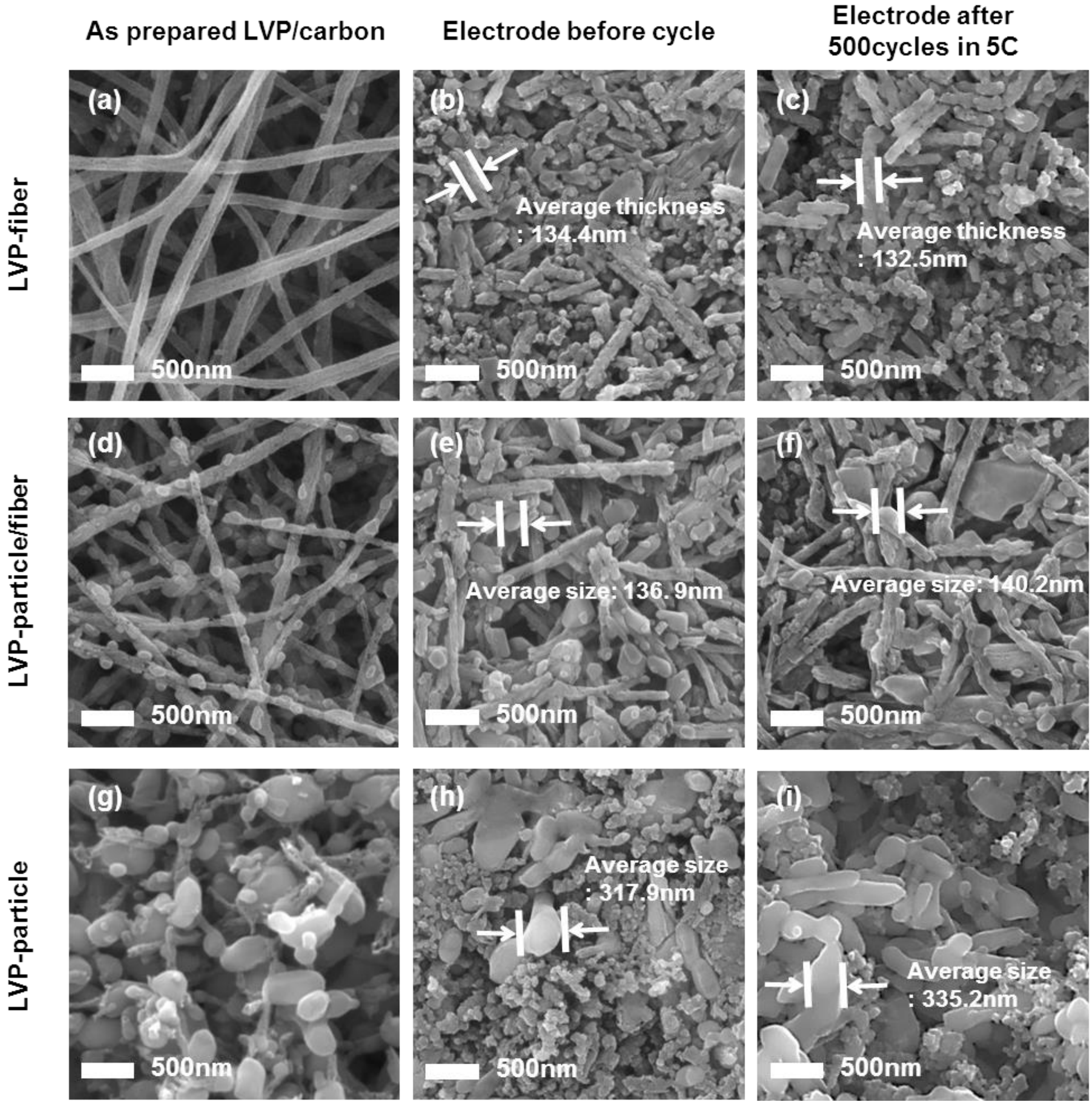

Figure S9. SEM images of LVP/carbon nanofiber composite (a, d, g) as prepared, (b, e, h) before cycling, and (c, f, i) after 500 cycles at 5C

Sample	R_e (mΩ)	R_f (Ω)	R_{ct} (Ω)
**LVP- fiber**	83.29	1.308	489.50
**LVP- particle/fiber**	96.04	1.224	68.72
**LVP- particle**	128.03	1.284	205.90

Table S2. Resistances of LVP/carbon nanofiber composites obtained from EIS spectra fitting with Z view software.

Additional experiments were conducted in order to see how temperature can affect the morphological characteristics. From here, temperature-controlled samples will be introduced. During the experiment, each sample was annealed at 600, 700, 800, 900 °C for 4 hrs.

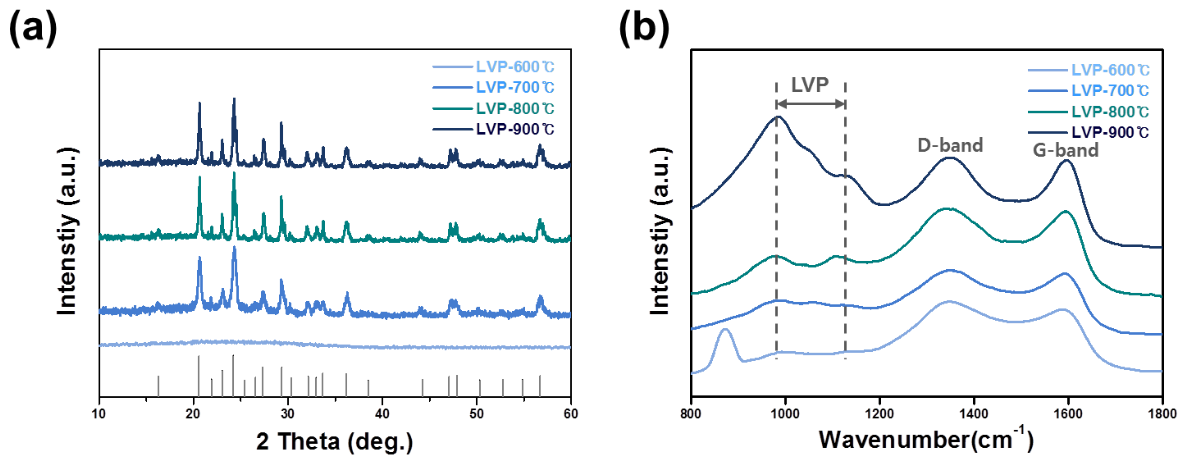

Figure S10. (a) XRD diffraction patterns and (b) Raman spectra of LVP/carbon nanofiber composites annealed at different temperatures.

In Raman spectra, the additional peak near 900 cm^{-1} is due to the amorphous $V_2(PO_4)_3$, which is the intermediate product before formation of crystalline $Li_3V_2(PO_4)_3$.

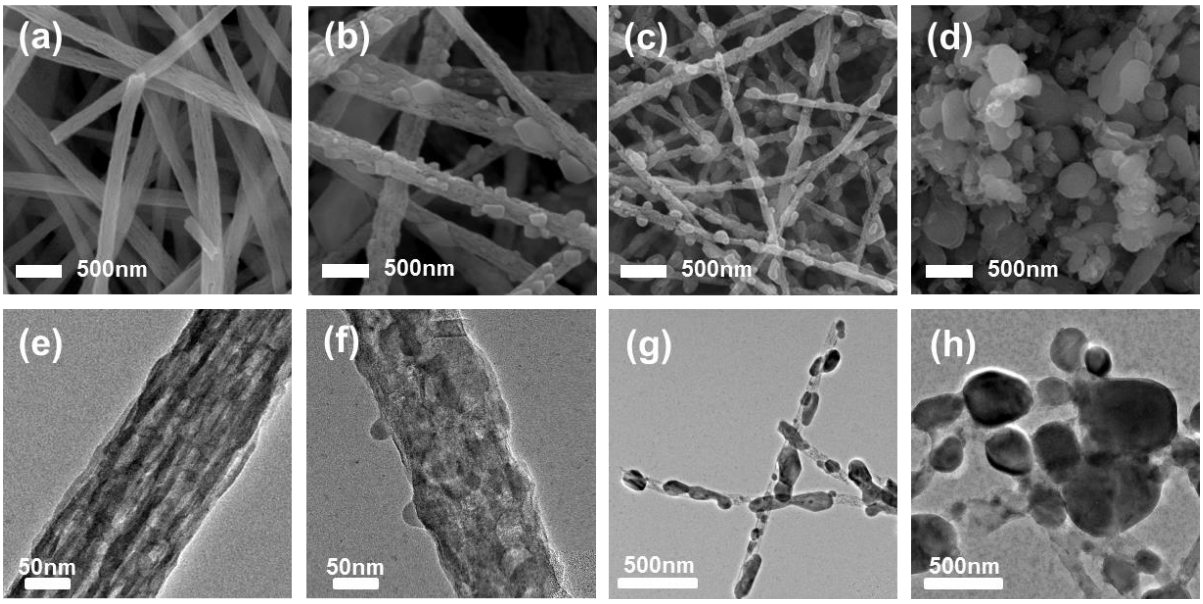

Figure S11. SEM images of LVP/carbon nanofiber composites synthesized at (a) 600°C, (b) 700°C, (c) 800°C, and (d) 900°C. TEM images of LVP/carbon nanofiber composites synthesized at (e) 600°C, (f) 700°C, (g) 800°C, and (h) 900°C.

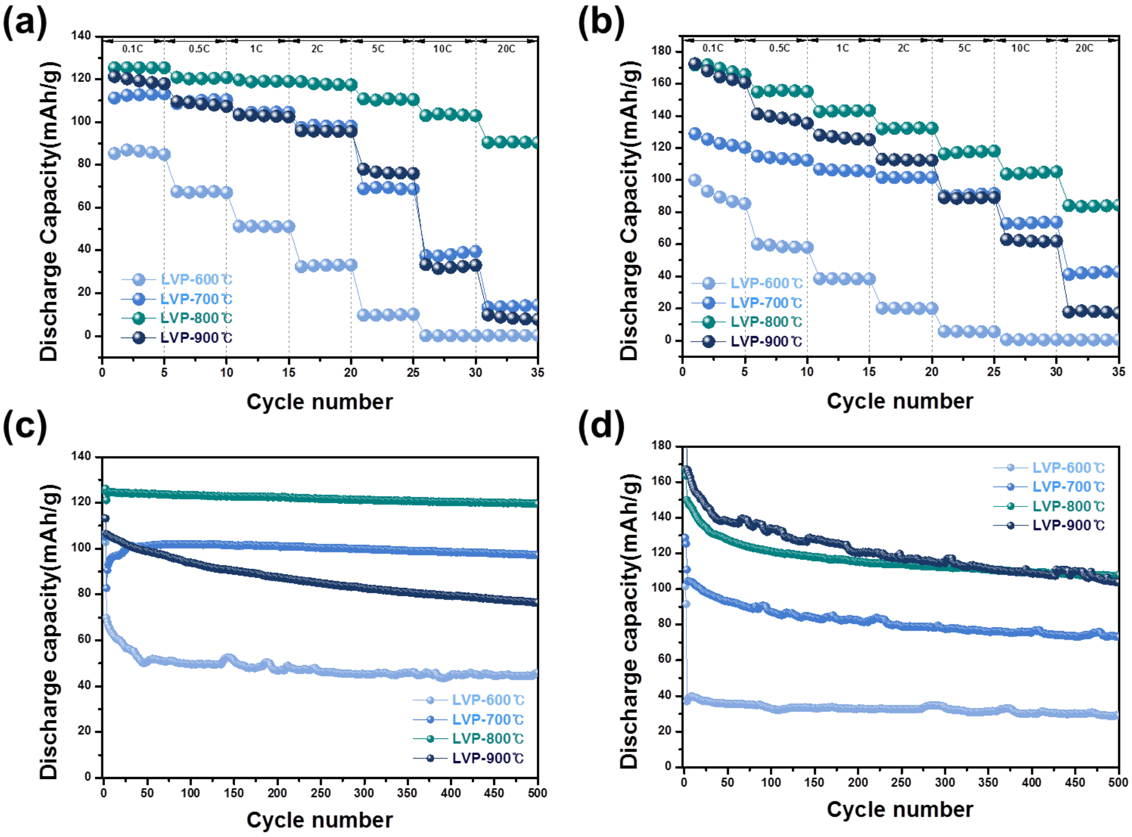

Figure S12. Rate capabilities of LVP/carbon nanofiber composites synthesized as different temperatures with the variation of C-rate from 0.1 to 20 C (a) between 3.0 – 4.3 V and (b) between 3.0 – 4.8 V (*vs.* Li^+/Li). Cyclic stabilities of these LVP/carbon nanofiber composites obtained at 0.1 C for first two cycles and 1 C for the following 500 cycles (c) between 3.0 – 4.3 V and (d) between 3.0 – 4.8 V (*vs.* Li^+/Li).
